# Patients with Prior Craniectomy or Craniotomy Have No Increased Risk of Acute Hemorrhage after Mild Traumatic Brain Injury

**DOI:** 10.3390/ijerph19052684

**Published:** 2022-02-25

**Authors:** Harald Binder, Daniel Schallmeiner, Thomas M. Tiefenboeck, Stephan Payr, Markus Winnisch, Richard Kdolsky, Stefan Hajdu, Gilbert Manuel Schwarz, Marcus Hofbauer

**Affiliations:** 1 Department of Orthopedics and Trauma Surgery, Medical University of Vienna, Waehringerguertel 18-20, 1090 Vienna, Austria; daniel.schallmeiner@gmail.com (D.S.); thomas.tiefenboeck@meduniwien.ac.at (T.M.T.); stephan.payr@meduniwien.ac.at (S.P.); richard.kdolsky@meduniwien.ac.at (R.K.); stefan.hajdu@meduniwien.ac.at (S.H.); gilbert.schwarz@meduniwien.ac.at (G.M.S.); marcus.hofbauer@meduniwien.ac.at (M.H.); 2 Department of Orthopaedics, Sacred Heart Hospital of Vienna, 1090 Vienna, Austria; markus.winnisch@kh-herzjesu.at

**Keywords:** traumatic brain injury, decompressive craniectomy, craniotomy, anticoagulation, outcome

## Abstract

The aim of this study was to retrospectively evaluate the risk of acute hemorrhagic complications in patients after either a decompressive craniectomy or a craniotomy sustaining a recurrent mild traumatic brain injury. Furthermore, we analyze whether there is a higher risk for acute hemorrhagic complications considering patients with anticoagulation compared with patients without anticoagulation in both groups. All patients with mild traumatic brain injuries after either decompressive craniectomy or craniotomy, treated between January 2005 and December 2020 at a single level 1 trauma center, were included in this retrospective analysis. Patients were screened for intracranial bleeding after mild traumatic brain injury with computed tomography. Additionally, the type of anticoagulation and its relationship concerning the clinical outcome were assessed. A total of 188 patients who had sustained a mild traumatic brain injury were included in the study. Overall, 22 patients (11.7%) presented intracranial lesions. A bony defect (decompressive craniectomy) was present in 31 patients (16.5%). In 157 patients (83.5%) who underwent decompressive craniectomy, the bony defect was closed during a second operation. There was no significant correlation between both groups on the occurrence of intracranial bleeding (*p* = 0.216). Furthermore, no difference was present between patients with and without anticoagulation (*p* = 0.794) concerning acute hemorrhagic complications after recurrent traumatic brain injury. Pre-existing bony defects after decompressive craniectomy showed no higher risk for acute hemorrhagic complications after recurrent mild traumatic brain injury compared with patients who primarily underwent craniotomy. Anticoagulation did not influence the occurrence of intracranial bleeding after mild traumatic brain injury in patients with decompressive craniectomy.

## 1. Introduction

Traumatic brain injury (TBI) affects 235 people per 100,000 every year, and is responsible for the majority of trauma-related deaths in Europe [1,2,3,4]. Even mild forms of trauma can lead to a wide range of secondary complications, including permanent restrictions on the cognitive abilities of patients [1], sleep complaints, anxiety and excitement, outbursts of anger and impulsiveness [5]. Although the number of patients with TBI aged <40 years is decreasing because of many safety precautions, a rise in geriatric patients with TBIs is present [4].

Falls are the main cause of TBIs in childhood and in seniors [6,7]. Traffic accidents and violent offenses are the main focus in young adults [6]. Sustaining a traumatic brain injury can lead to an intracranial hemorrhage [8] or a secondary increase in intracranial pressure [9] which may require a surgical intervention. Surgical procedures to open the intracranial space [10] include a decompressive craniectomy or a craniotomy. The difference between these two interventions is that during a decompressive craniectomy, a part of the skull is removed. A skull defect can remain, or it can be covered with a secondary operation [11]. During a craniotomy, the removed bone flap is replaced at the end of the same intervention [12]. A considerable number of patients with acute TBI present a pre-existing condition after a craniotomy or decompressive craniectomy.

Particularly in the case of craniectomy, when secondary coverage of the bony defect has not yet been performed, a larger area of the brain is relatively unprotected beneath the skin and muscle flap. This suggests that these patients sustaining a recurrent TBI are at a higher risk of intracranial bleeding compared with patients with an intact skull. There are numerous studies regarding the effectiveness/outcomes of craniotomy or decompressive craniectomy after a TBI with the occurrence of intracranial bleeding (ICB). In particular, decompressive craniectomy is an often-investigated surgical method, with numerous clinical studies having been conducted [12,13,14,15]. However, the risk of an ICB in a patient with recurrent TBI and a pre-existing craniotomy/craniectomy has not yet been investigated, and there are no comparable data in the literature.

The aims of this study were to analyze the risk of acute hemorrhagic complications after a new fall in patients treated surgically for ICB, and analyze the risk of intracranial bleeding (ICB) in such patients with current anticoagulation. 

## 2. Materials and Methods

### 2.1. Data Collection

Data for this study were obtained retrospectively from the records of patients that already had either a decompressive craniectomy or a craniotomy and were admitted with recurrent TBI to a level 1 trauma center between January 2005 and December 2020. Inclusion criteria were conditions after a craniectomy with a bony defect or a craniotomy without a defect and craniectomy with a closed defect (date of surgical procedure as well as known CT scans). The implemented procedure was identified based on the initial CT scan assessments and radiological reports. In terms of secondary closure concerning bony defects, the implants used, including autologous bone, were recorded. Data on the demographic characteristics of patients (age, sex), cause of injury, Glasgow Coma Scale (GCS), CT findings, and treatment modalities (surgical vs. conservative, types of surgery, additional treatment) were recorded. In order to distinguish a pre-existing craniotomy from a craniectomy with secondary coverage by autologous bone implantation, information from surgery reports or the patient’s medical history was used. The severity of acute TBI was determined using the GCS. GCS values of 13 to 15 correspond to a mild TBI; GCS values of 9 to 12 correspond to a moderate TBI; and GCS values of 3 to 8 correspond to a severe TBI. 

### 2.2. Treatment Procedures in TBI Patients Admitted to Our Ward

#### 2.2.1. First-Line Therapy

All admitted patients were examined by an emergency physician, including assessment of the GCS documentation, pupillary reactivity and the current coagulation state. Further medical treatment consisted of rapid-sequence intubation, ventilation, treatment of hemorrhage, treatment of associated substantial extra cranial injuries and fluid resuscitation, if required.

#### 2.2.2. Radiological Assessment and Continued Treatment

Each patient underwent a CT examination and an examination by a trauma team (consisting of trauma surgeons, radiologists, anesthesiologists and/or neurosurgeons and specialized nurses). Depending on the results of the CT, patients underwent surgery and/or were admitted to the ICU or a normal ward. In complex cases, neurosurgeons additionally consulted with trauma surgeons. Intensive care treatment was provided by anesthesiologists in cooperation with trauma surgeons and neurosurgeons. 

Prehospital parameters and treatment were documented by paramedics. CT scan findings were interpreted by trauma surgeons, neurosurgeons, and radiologists. Data regarding the duration of various treatments, complications and outcomes were collected at hospital discharge and at follow-up.

#### 2.2.3. Postoperative Treatment Protocol

Our postoperative treatment protocol consists of upper-body elevation at an angle of 30–45°. Intravenous mannitol and hypertonic saline are routinely used to control intracranial hypertension after or concurrently with sedation and mild hyperventilation. Sedation takes place with benzodiazepines or propofol. For analgesic effects, opioids are preferably used. Invasive intracranial pressure (ICP) measurements are required for the continuous determination of cerebral perfusion pressure (CPP), which should not fall below 50 mmHg nor be raised above 70 mmHg by aggressive therapy.

The measurement takes place via external ventricular drainage which, especially in patients with severe intraventricular hemorrhage (IVH) or deep hematomas indicates important intraventricular invasion as well as the progressive worsening of levels of consciousness. Ventricular drainage is removed before the patient is transferred to a normal ward or in a standardized manner when conditions are stable with normal CPP values. A standardized antibiotic therapy is performed as well as an anticonvulsant therapy to prevent a seizure during the first week. For prognostic purposes, and in addition to the routine parameters, we documented the hemoglobin levels and blood glucose concentrations upon admission and after 24 h in all patients. CT scans are performed immediately postoperatively, 24 h and 48 h after surgery. Subsequent checks are only performed in cases of neurological deterioration. The quickest possible initiation of a neurological–neurosurgical rehabilitation measure according to the severity of the dysfunction should be strived for, which is why physical therapy measures are carried out in the intensive care unit tailored to the patient.

#### 2.2.4. Follow-Up Examination

After discharge from the hospital follow-up, examinations are performed at regular intervals of 4 weeks, 12 weeks and 6 months, and then annually, depending on the clinic. This is also in accordance with the guidelines of craniocerebral trauma treatment in adulthood, developed by the German Society for Neurosurgery (DGNC).

### 2.3. Data Analysis

In all patients’ demographic factors, injury causes, trauma characteristics, anticoagulation medication intake and severity were compared. Patients were assigned either to the “craniectomy” group or “craniotomy” group if they underwent one of the procedures before their administration to the hospital concerning a recent TBI.

The statistical analysis included a tabular description of the data. Means and standard deviations were calculated for metric data and nominal data frequencies, and percentages are shown. SPSS software version 24.0 (SPSS Inc., IBM, Chicago, IL, USA) was used to perform the statistical evaluation. Groups were compared using Fisher’s exact test. Statistical tests were considered statistically significant when *p*-values were lower than 0.05.

## 3. Results

### 3.1. Patients

**Figure 1:**
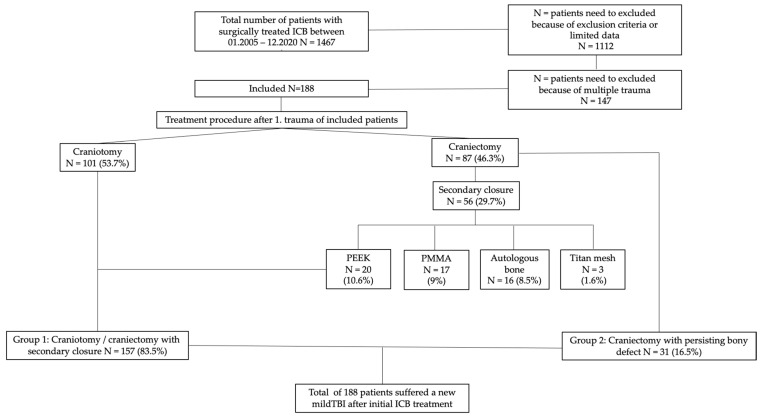
Patient flowchart.

A total of 188 patients (56.9% male; 43.1% female) were included in this study. There were a total of 157 patients (83.5% of all patients) who originally underwent decompressive craniectomy or craniotomy. Detailed information of included patients is presented in the patient flow chart (Figure 1).

In 50% of the patients who underwent decompressive craniectomy, the bony defect was closed during a second operation. Regarding this type of surgical procedure, secondary coverage was provided by the patient’s preserved autologous bone material, Polyaryletherketone (PEEK), Palacos^®^ bone cement (PMMA = Polymethylmetracrylat), or by a titanium mesh.

#### 3.1.1. Repeat Mild TBI

The average age at the time of the recent trauma was 55.6 ± 18 years, with a mean of 8.5 ± 10.9 years concerning the interval of the surgical procedure. In 164 patients (87.2%), the trauma was defined as recent, implying that the cause that led to the trauma was not longer than 24 h ago. In 24 patients (12.8%), the trauma was longer than 24 h ago. A total of 142 patients (75.5%) showed obvious signs of skull injuries, 9 patients (4.8%) presented a fracture in this area, 22 (11.7%) showed intracranial bleeding and 4 patients (2.1%) needed rapid-sequence intubation. Detailed results are presented in Table 1.

#### 3.1.2. Treatment Factors in Surgically Managed Patients

Analogous to the classification of the TBI [13], patients were assigned to three different severity levels based on the GCS values. Most (92%) of the patients examined presented with mild TBI.

Surgical intervention was necessary in 7 patients (3.7%). In 3 patients (1.6%) a craniotomy was performed, in 4 patients (2.1%) a decompressive craniectomy was conducted, and in 1 patient (0.5%) a burr hole trepanation was carried out. A lethal outcome was found in one patient (0.5%). Alcohol intoxication led to a fall and thus to acute TBI in 25 patients (13.3%).

#### 3.1.3. Therapeutic Anticoagulation

A total of 45 patients (23.9%) had a therapeutic inhibition of blood coagulation at the time of acute TBI. In 6 (3.2%) patients, no information could be found in the retrospective data. The remaining 137 patients (72.9%) did not take any anticoagulant drugs. The frequencies of all observed drugs are shown in Table 2.

#### 3.1.4. Bleedings after Repeated TBI

A total of 22 ICBs (11.7%) were detected; in 8 (3.7%) patients, multiple bleedings occurred. The following bleedings were found: 7 epidural hematoma, 8 subdural hematoma, 7 subarachnoidal hematoma and 12 intracerebral bleedings. 

There was no significant correlation between patients with or without anticoagulation (*p* = 0.794) concerning the acute hemorrhagic complications found.

Comparing the two different groups, no significance regarding the intracranial hemorrhages was found (*p* = 0.216).

## 4. Discussion

Traumatic brain injury (TBI) is the most common cause of death and disability in those aged under 40 years. Although the rates of morbidity and mortality are reduced in Western countries, it remains high in low-income and middle-income countries, making it a global health challenge [13]. Yearly, TBI costs the global economy approximately USD 400 billion, representing 0.5% of the gross world product [13].

Severe TBI presents with a mortality rate of 30–40% and can cause significant physical, psychosocial, and social deficits in up to 60% of cases [14]. Therefore, taking a closer look at repeated trauma after the surgical treatment of TBI is of importance. 

To the best of our knowledge, this is the first study in the literature taking a closer look at patients sustaining a mild TBI after a craniotomy or craniectomy. In summary, the results did not show any significant difference regarding the rate of bleeding after TBI according to a low-energy trauma between patients undergoing a craniectomy or craniotomy. Additionally, the coagulation status did not lead to a higher number of bleedings following mild TBI after surgically treated ICBs. 

Mild TBI is the main diagnosis for head trauma, occurring at a rate of 91.3%, indicating the importance of this injury [4]. Mauritz et al. demonstrated males to be more common in mild TBI [4]. This was also reflected in our data. However, patients in our study presented as generally older (a mean of 56 years compared with a mean 41 years) compared with similar studies [15]. 

The second hypothesis of the study could also not be confirmed. An anticoagulant medication was not correlated with a higher rate of hemorrhage in either the craniotomy or the craniectomy group. Traumatic intracranial hemorrhage was reported to occur more frequently in patients with anticoagulation therapy, ranging from 0.2% to 6% [16,17,18,19]; however, the presented study could not show significancy. 

A recent study by Antoni et al. (2019) retrospectively analyzed the data of patients with head trauma and antithrombotic therapy without pathologies on their initial CT. A total of 390 patients received routine repeat CT scans and showed 9 delayed hemorrhages (2.3%) [20]. This rate is similar to that presented in this study with 8 cases (2.7%), showing intracranial bleeding.

In accordance with the above mentioned study by Antoni et al. (2019) [20], we also do not see any necessity of routinely repeating CT scans without neurological deterioration if patients are observed in a clinical setting. This applies to both groups; however, there are some authors who even question the necessity for clinical observation after negative CT scans [21,22,23]. Contemporary treatment algorithms of TBI are not consistent, and randomized controlled trials are lacking to close this gap [13].

### Limitations

There are considerable limitations to this study. These resulted as a consequence of the retrospective study design. In a prospective study with the question of bleeding risk in pre-operated patients, more significant results could be obtained. However, this would involve a year-long recruitment process of patients in order to obtain an adequate number of cases. However, this study represents a homogeneous patient group with the main outcome parameters reported, and is therefore able to draw valid conclusions.

## 5. Conclusions

Pre-existing bony defects after decompressive craniectomy showed no higher risk for acute hemorrhagic complications after recurrent mild traumatic brain injury compared with patients who primarily underwent craniotomy. Anticoagulation did not influence the occurrence of intracranial bleeding after mild traumatic brain injury in patients with decompressive craniectomy.

## Figures and Tables

**Table 1:** Comparison of patient’s demographics in detail.

Craniectomy with Bony Defect (*n* = 31)		Craniotomy/Craniectomy with Secondary Closure (*n* = 157)	*p*-Values
Age in years			0.211
59.02 ± 17.81		56.03 ± 10.7	
Sex			0.836
male: 20 (64.5%)		male: 105 (66.9%)	
female: 11 (35.5%)		female: 52 (33.1%)	
Anticoagulation			0.794
yes: 10 (32.3%)		yes: 35 (22.3%)	
no: 21 (67.7)		no: 122 (77.7%)	
Trauma mechanism			
Mild TBI: 31 (100%)		Mild TBI: 157 (100%)	
Bleedings			0.216
6 (19.4%)		16 (10.2%)	
SDB: 2 (6.5%)		SDB: 6 (3.8%)	
EDH: 1 (3.2%)		EDH: 6 (3.8%)	
ICB: 4 (12.9%)		ICB: 8 (5.1%)	
SABL: 4 (12.9%)		SABL: 3 (1.9%)	
GCS			0.042
3–8: 4 (12.9%)		3–8: 5 (3.2%)	
13–15: 27 (87.1%)		13–15: 152 (96.8%)	
Treatment necessary	6 (100%)	16 (100%)	
Surgical treatment			1.000
2 (33.3%)		5 (31.3%)	
Conservative treatment			
4 (66.6%)		11 (68.7%)	

**Table 2:** Frequency distribution of anticoagulant drugs.

Type of Anticoagulation	Frequency	Percentage
T-Ass, Plavix, (Brillique)	28	14.9
Ivor, Fragmin, Lovenox	12	6.4
Marcoumar, Sintrom	7	3.7
Xarelto, Pradaxa, Lixiana	7	3.7
Total	54	28.7

## Data Availability

The data presented in this study are available on request from the corresponding author. The data are not publicly available due to privacy.
